# Covariation Analysis of Serumal and Urinary Metabolites Suggests Aberrant Glycine and Fatty Acid Metabolism in Chronic Hepatitis B

**DOI:** 10.1371/journal.pone.0156166

**Published:** 2016-05-26

**Authors:** Linlin Yang, Xue Yang, Xiangliang Kong, Zhiwei Cao, Yongyu Zhang, Yiyang Hu, Kailin Tang

**Affiliations:** 1 School of Life Science and Technology, Tongji University, Shanghai, China; 2 Changhai hospital of traditional Chinese Medicine, Second Military medical university, Shanghai, China; 3 Shanghai University of Traditional Chinese Medicine, Shanghai, China; 4 Institute of Liver Diseases, Shuguang Hospital, Key Laboratory of Liver and Kidney Diseases of Ministry of Education, Shanghai University of Traditional Chinese Medicine, Shanghai, China; 5 Advanced Institute of Translational Medicine, Tongji University, Shanghai, China; University of North Carolina at Charlotte, UNITED STATES

## Abstract

**Background:**

Chronic hepatitis b (CHB) is one of the most serious viral diseases threatening human health by putting patients at lifelong risk of cirrhosis and hepatocellular carcinoma (HCC). Although some proofs of altered metabolites in CHB were accumulated, its metabolic mechanism remains poorly understood. Analyzing covariations between metabolites may provide new hints toward underlying metabolic pathogenesis in CHB patients.

**Methods:**

The present study collected paired urine and serum samples from the same subjects including 145 CHB and 23 healthy controls. A large-scale analysis of metabolites’ covariation within and across biofluids was systematically done to explore the underlying biological evidences for reprogrammed metabolism in CHB. Randomization and relative ranking difference were introduced to reduce bias caused by different sample size. More importantly, functional indication was interpreted by mapping differentially changed covariations to known metabolic pathways.

**Results:**

Our results suggested reprogrammed pathways related to glycine metabolism, fatty acids metabolism and TCA cycle in CHB patients. With further improvement, the covariation analysis combined with network association study would pave new alternative way to interpret functional clues in clinical multi-omics data.

## Introduction

Since being discovered in 1967, hepatitis B virus (HBV) infection has become one of the most serious viral diseases threatening human health, particularly in developing countries [1]. Patients positive for hepatitis B surface antigen (HBsAg) for more than 6 months will be diagnosed as chronic hepatitis b (CHB) [2], which dominates the population infected with HBV in China [3]. Persistent infection of HBV would not only cause inflammation in liver, but, more importantly, put patients at lifelong risk of cirrhosis and hepatocellular carcinoma (HCC) [4].

As liver is a vital metabolic organ for human, many efforts from metabolomics researchers have been devoted to monitor patients’ changes in metabolic profiles in order to probe the pathogenesis of HBV-induced liver diseases. By analyzing nuclear magnetic resonance spectroscopy (NMR) data, Tang et al. showed that HBV infection could induce hexosamine, choline and central carbon metabolism alteration in HepG2.2.15 cell model [5]. Makoto et al. applied liquid chromatography electrospray tandem mass spectrometry (LC-MS) to analyze the serum from 14 CHB patients and proposed γ-glutamyl threonine as a feature to discriminate CHB from other liver diseases [6]. Further, based on 82 HCC patients’ paired serum and urine metabolic profiles, Jia et al. identified 4 metabolites as potential markers for clinical stratification of HCC [7].

Although the above insightful studies have shown the usefulness of metabolomics in identifying relevant chemical markers for liver diseases, they mainly focused on the changes of individual metabolites between different physiological states. It is noted that liver dysfunction would disrupt the homoeostasis in form of altered metabolic flux in patients with liver diseases. Thus, instead of individual compound, analyzing covariations between metabolites [8] may provide new hints to those aberrant pathways so as to highlight the underlying metabolic pathogenesis in CHB patients.

In the present study, urine and serum samples were collected from the same subjects at the same time. A total of 168 paired serum and urine profiles were obtained from 85 CHB patients and 23 healthy individuals via gas chromatography-mass spectrometer (GC-MS). A large-scale covariation analysis of metabolites within and across bio-fluids was systematically done to explore the underlying biological evidences for reprogrammed metabolism in CHB. Our results implied that glycine and fatty acids metabolism may be reshaped in CHB patients.

## Materials and Methods

### Subject selection and sampling

85 CHB patients and 23 healthy controls were enrolled in this study. All CHB patients were at the age of 18 to 65 years old and must accord with the diagnostic criteria for CHB [9,10]: a) persistence of HBsAg for more than six months or b) with history of HBV infection (≥ 6 months) and still positive for HBsAg. Each patient must show a) persistent or recurrent elevation of serum alanine aminotransferase (ALT) or b) hepatitis lesions revealed by ultrasonic examination. Meanwhile, healthy volunteers without HBV infection were recruited as control. Any CHB or healthy subjects with other hepatotropic virus infection, chronic severe hepatitis and other serious disease primarily occurring in heart, kidney, lung, endocrine, blood, metabolism and gastrointestinal tract were excluded during sample selection. Psychiatric patients, pregnant or lactating women were excluded as well. Our study was approved by the IRB of Shuguang Hospital affiliated with Shanghai University of TCM and conducted according to the principles expressed in the Declaration of Helsinki. The use of these subjects was approved by the hospital’s Ethics Committee and all participants provided their written informed consents.

In CHB group, some of the CHB patients were continuously monitored every 3 months: 17 were additionally monitored twice and 26 were additionally monitored once with a three months interval after the first sampling. Besides, 23 healthy volunteers were recruited as control. Thus, 145 CHB samples and 23 healthy individuals were included in this study. Morning urine and fasting serum were collected from all enrolled subjects and immediately stored at -80°C until GC-MS assay.

### Sample preparation and data acquisition

The collected samples were prepared and processed using GC-MS as previously described [11] except the serum sample preparation and the column temperature program.

1. Serum sample preparation. 100μL of each serum sample was transferred into a screw tube. After adding 10μL L-2-Chlorophenylalanine, 10μL Heptadecanoic acid, 300ul Solvent (Methanol: Chlorform, 3:1, V:V) and vortex-mixing for 30s, samples were conditioned at -20°C for 10min to precipitate protein. Then the solution was centrifuged at 13,000 rpm for 10min.
2. Column temperature program. The temperature program of column incubator used in our GC-MS was listed in S1 Table.

### Metabolites identification

After all the GC-MS raw files were converted to CDF format via the software with Agilent MSD workstation, peak alignment was performed subsequently by a package named “xcms” in R [12]. Default settings for xcms was: xcmsSet (full width at half-maximum: fwhm = 5; S/N cutoff value: snthresh = 10, max = 25), group (bw = 5). The obtained area for each peak was natural logarithmic transformed and normalized as equation below.

$$\text{Normalized}\left(P_{i,j}\right)=\;\frac{P_{i,j}-\text{min}\left\{P_{1,j}\ldots P_{n,j}\right\}}{\text{max}\left\{P_{1,j}\ldots P_{n,j}\right\}-\text{min}\left\{P_{1,j}\ldots P_{n,j}\right\}}$$ (1)

Where, P_*i*,*j*_ is the natural logarithmic transformed peak area of compound *i* in sample *j*, and *n* is the total number of metabolites detected in the corresponding metabolic profile.

To identify metabolites in bio-fluids, we searched each sample’s spectra data against NIST 2005 database in Agilent MSD workstation. Spectra patterns with matching similarity higher than 80% were recognized as chemicals. Then the identified chemicals were subsequently aligned with peaks identified by “xcms” according to their retention time. If several peaks simultaneously showed retention time approaching one metabolite (±0.03 min), the sum of these peaks were used to quantify the corresponding metabolite.

### Network analysis

#### Randomization for CHB

In this study, the numbers of samples we collected for each group were not equal. There were 145 samples in CHB, but only 23 samples in healthy group. Imbalanced sample size may cause different distribution of correlations and hence brought bias to comparison of CHB and healthy group. Thus, for each compound pair in CHB group, we simulated its distribution in condition of small sample size. 23 samples (equal to the number of healthy group) were randomly selected from CHB group. Considering there were multiple time points in CHB group, we excluded those random sets with multiple samples corresponding to one person. Totally, 500 random sets without individual redundancy were obtained and considered as randomization for CHB. By doing this, covariation in CHB group was regarded to be comparable to that in healthy group.

#### Measurement of covariation

The present study used Spearman’s rank correlation to measure the covariation between metabolites. As no significant difference of age, gender and BMI was found between CHB and healthy group, adjustment of covariations was skipped in this paper.

For given metabolites A and B in a group with *n* samples:

$$\text{A}=\left(a_{1},a_{2},\ldots ,a_{i},\ldots a_{n}\right)$$ (2.1)

$$\text{B}=\left(b_{1},b_{2},\ldots ,b_{i},\ldots b_{n}\right)$$ (2.2)

the correlation between them (*Cor*_*A*,*B*_) can be calculated as:

$$Cor_{A,B}=1-\frac{6\;{\sum^{\;}}^{\;}d_{i}^{2}}{n\left(n^{2}-\;1\right)}$$ (3)

$$d_{i}=\text{Rank}(a_{i})-\text{Rank}(b_{i})$$ (4)

Where, *a*_*i*_ is the normalized peak area of metabolite A in sample *i*; *b*_*i*_ is the normalized peak area of metabolite B in sample *i*; *d*_*i*_ is the sample *i*‘s rank difference between metabolite A and B in the given group. If A and B belong to the same biofluid, their correlation can be classified as intra-covariation; if else, inter-covariations.

#### Differentially covaried metabolic network for CHB

The differentially covaried metabolic network was designed to provide biological indication for altered metabolism in CHB. It was constructed by covariations significantly changed between CHB and healthy group. The evaluation of differential covariations was performed by using two parameters: Z-score and rank difference (*d*_*rank*_).

For one compound pair, its Spearman correlations in healthy group (*Cor*_*health*_) and CHB simulations (*Cor*_*rand*,*1*_, …, *Cor*_*rand*,*500*_) were calculated. Subsequently, its average value ($\bar{Cor_{random}}$) and standard deviation (*σ*_*random*_)in the 500 CHB random sets could be obtained. Then Z-score [13] was calculated to measure its significance of difference between CHB and healthy group:

$$\text{Z}-\text{score}=\;\frac{\bar{Cor_{random}}-Cor_{health}}{\sigma_{random}}$$ (5)

Additionally, the rank difference (*d*_*rank*_) of one compound pair (*Cor*_*A*,*B*_) between CHB and healthy controls was considered.

$$d_{rank}=\left|\text{Rank}{\left(Cor_{A,B}\right)}_{\text{CHB}}-\text{Rank}{\left(Cor_{A,B}\right)}_{\text{health}}\right|$$ (6)

Where, Rank(*Cor*_*A*,*B*_)_CHB_ represents the rank of *Cor*_*A*,*B*_ among all the 11175 compound pairs in CHB group (n = 145), and Rank(*Cor*_*A*,*B*_)_health_ represents the rank of *Cor*_*A*,*B*_ among all the 11175 compound pairs in healthy control (n = 23).

Here, correlations simultaneously reaching two thresholds would be recognized as differential covariations: (1) |Z| > 3, which means the difference between healthy people and CHB patients reaches to a significant level, and (2) *d*_*rank*_ > 7000, which corresponds to TOP 3% on the covariation list (11175 pairs) with large rank differences.

### CHB sample stratification

For each sample enrolled (CHB and health), clinical data including age, sex, and body mass index (BMI) were recorded. Serological tests of HBV DNA, alkaline phosphatase (ALP), alanine aminotransferase (ALT), aspartate transaminase (AST), gamma-glutamyl transpetidase (GGT) and total bile acid [2] were immediately performed by hospital’s clinical laboratory after sampling. Here, to monitor metabolic alterations across different grade of CHB, we stratified the CHB samples according to their liver function parameters and HBV DNA copy number:

1. For liver function, CHB samples with any parameter (ALT, AST, ALP or GGT) out of normal range were assigned to the liver dysfunction group (n = 100); and others with all parameters (ALT, AST, ALP and GGT) in normal range were assigned to mild CHB group (n = 45).
2. As to HBV DNA load, a parameter indicating viral replication, CHB samples with HBV DNA > 1000 copies/mL were assigned as high copy group (n = 100); otherwise, low copy group (n = 37).

Levels of all urinary and serumal metabolites were compared between different CHB subgroups (student’s t test, two tailed).

### Gene expression profiles

To search for enzymatic clues for the metabolic alterations suggested by our covariation analysis, we screened two gene expression profiles from: a) peripheral blood mononuclear cell (PBMC) in 5 healthy individuals and 12 CHB patients (GSE58208, http://www.ncbi.nlm.nih.gov/geo/query/acc.cgi?acc=GSE58208) and b) liver tissues in woodchuck model of CHB including 30 chronically infected animals and 60 uninfected ones (GSE36533) [14]. For each dataset, gene expression in CHB group and healthy controls was compared. Genes showed P-value < 0.05 (student’s t test) and large log2 fold change (|logFC| > 0.5) were regarded to be significantly differentially expressed between CHB and healthy controls.

## Results and Discussion

### Overview of samples and metabolites

We analyzed the GC-MS metabolic profiles of 168 samples corresponding to 108 participants. The redundancy was described in method section. The basic information of all the non-redundant participants was recorded in Table 1. 69 and 81 metabolites were identified in serum and urine, which were listed in S2 and S3 Tables. Totally, 16 metabolites co-occurred in both serumal and urinary metabolic profiles of each sample.

**Table 1:** Demographic information of the Enrolled Population.

		CHB (n = 85)^§^	Health (n = 23)^§^
Age (years)		38 (17~64)	31 (21~58)
Gender (Male, %)		62 (72.9%)	16 (69.6%)
BMI (kg/m^2^)		22.23 (16.33~29.41)	21.08 (19.49~24.62)
HBV DNA (×10^4^cps/mL)^¶^		1.17 (0~25970)	—
ALT (IU/L)^¶^		48.53 (13~483.6)	17 (10~31)

^§^Data is presented as n (%) or median (min ~ max). A significant difference between CHB and healthy group exists if p-value < 0.05 (Wilcoxon rank sum test).

^¶^These clinical indicators in CHB group were calculated based on the 145 samples.

### Overall covariations across different samples

Results of intra-covariations within serum or urine metabolites and inter- ones between the two biofluids were summarized respectively for CHB patients and healthy individuals in S1 and S2 Figs. It can be seen that the correlations within urinary metabolites were higher than those within serumal ones, no matter in healthy people or CHB patients. This may be related to the inherent nature of serum as buffer, while the urine as terminal pool gathering excess materials, which makes urinary metabolites more inclined to vary jointly. In addition, the overall metabolic correlations within one bio-fluid were stronger than those between different bio-fluids in both CHB and healthy populations, as being expected.

**Fig 1:**
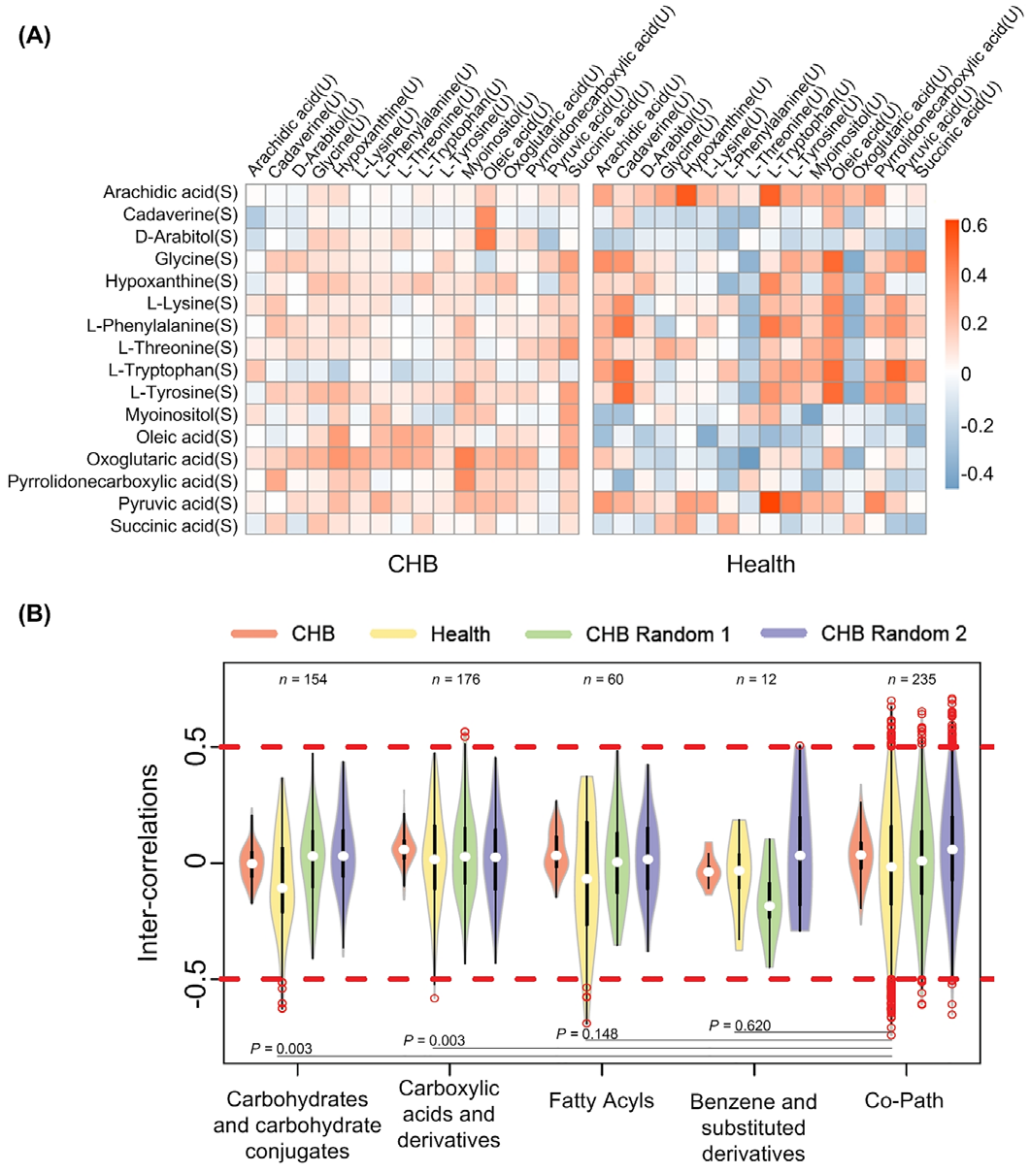
Inter-correlations between serumal and urinary metabolites. (A) Correlation between overlapped metabolites of serum and urine. “U” means compound in urine and “S” means metabolites in serum. Each lattice denotes correlation within one metabolite pair. (B) Distribution of inter-covariations between metabolites within the same category or pathway. Categories were identified based on the chemical taxonomy in HMDB [15]; only categories possessing more than 10 inter-covariations were displayed; the number of inter-covariations possessed by each category was shown in the top region; among the categories, differences of covariations’ distribution were calculated based on healthy group in form of P-values (Kolmogorov-Smirnov test); CHB random 1 and 2 are two CHB random sets with the same sample number as healthy controls.

Then we studied the inter-correlations between the 16 common metabolites shared by urine and serum. For CHB and healthy controls, inter-covariation for each pair of common metabolites was shown in Fig 1A. It can be seen that, comparing to healthy controls, the metabolites in CHB tended to show weaker correlations, which may be related to the large sample size in CHB group. For more detailed analysis, we further grouped our metabolites according to their chemical taxonomy and pathways. Fig 1B displayed the distribution of inter-correlations between metabolites within the same group. In Fig 1B, over 97% of the inter-covariations showed low intensity (correlation higher than 0.5 or lower than -0.5), suggesting weak correlation between serum and urine in both CHB and healthy status. Besides, more covariations with high intensity could be observed in co-pathway compound pairs than in co-class ones, especially when comparing to “Carbohydrates and carbohydrate conjugates” and “Carboxylic acids and derivatives” based on healthy individuals (P < 0.05, see Fig 1B). It was indicated that metabolites tended to be more correlated to each other when they were located in the same pathway.

So far, our results suggested a large difference between urine and serum metabolite profiles even from the same subject in both healthy and CHB population (S3 Fig). Moreover, seldom correlation was found between serum and urinary metabolites, especially in CHB group. Therefore, an integrative analysis is necessary for more comprehensive understanding of the metabolic aberration caused by HBV infection.

### Differentially co-varied network implies aberrant metabolic flux in CHB

**Fig 2:**
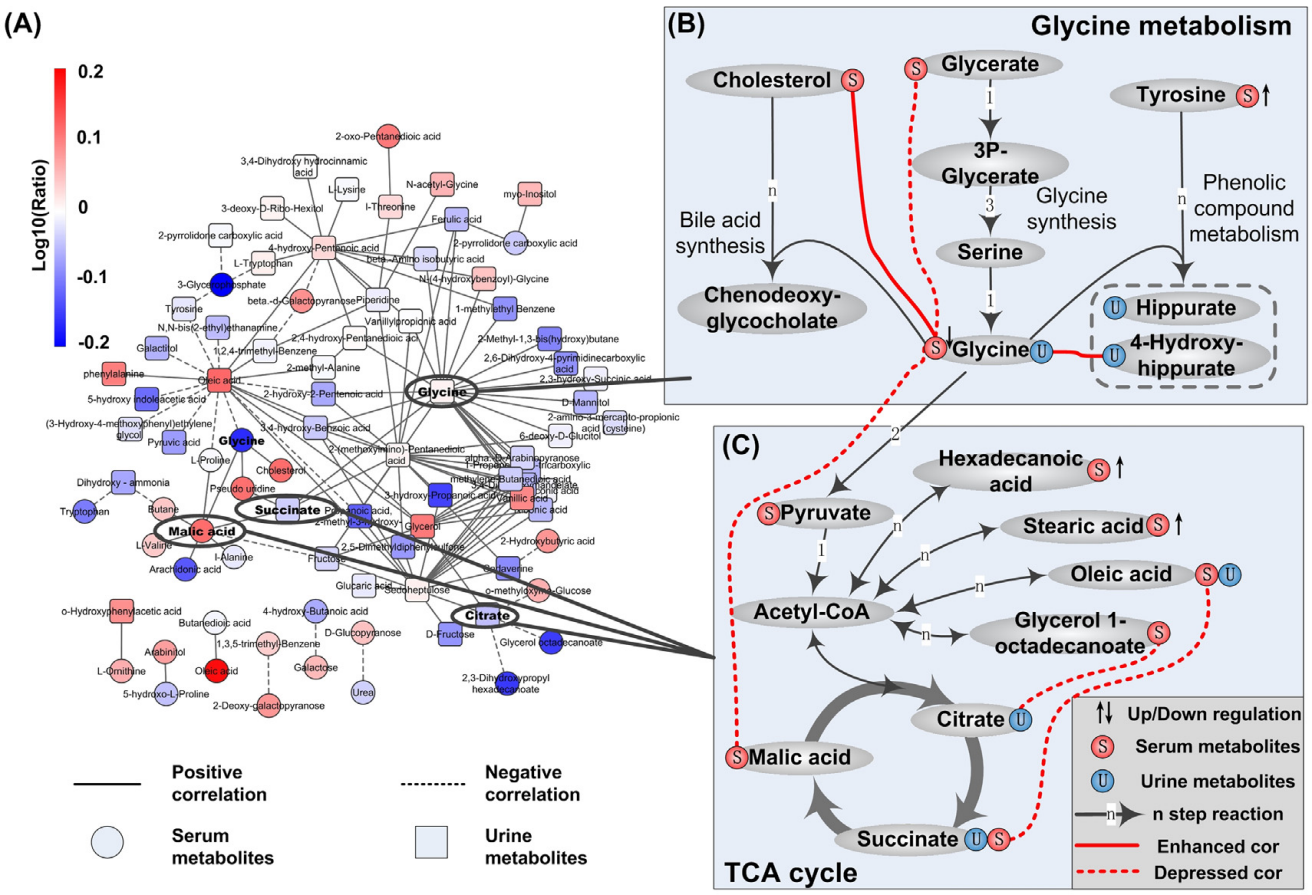
Abnormal metabolism indicated by differentially co-varied metabolic network of CHB. (A) Differentially co-varied metabolic network of CHB. Red nodes denote up-regulated metabolites in CHB group and blue nodes denote down-regulated ones. (B) Aberrant glycine metabolism in CHB. Edges with red color are significantly changed covariations in CHB. And compounds followed with up/down arrows are significantly differential metabolites. (C) Fatty acids metabolism connecting with TCA cycle.

Metabolites from urine and serum were pooled together for co-variation analysis. To reduce the bias caused by different sample size between CHB and healthy controls, we randomly select the same number of samples as healthy group from CHB subjects for 500 times. For each compound pair, correlation in healthy group and those in the 500 CHB randomizations were compared through Z-score. Additionally, to further reduce the influence from imbalanced sample size, we removed those pairs with large difference in correlation value but small relative ranking change between CHB and healthy groups. Totally, from background pool of 11,175 pairs, 130 significantly differential covariations (|Z| > 3 and *d*_*rank*_ > 7000) were identified between CHB patients and healthy individuals and subsequently used to construct the differentially co-varied network of CHB, which was displayed in Fig 2A. In this network, degree, the number of connections, held by each metabolite was screened. The top 10 hub metabolites were listed in S4 Table, where glycine was ranked as No. 1 and two metabolites were involved in TCA cycle. We then mapped those differential covariations around them to the known metabolic pathways, as shown in Fig 2B and 2C. Detailed information of differentially changed covariations and metabolites in Fig 2B and 2C was listed in Table 2.

**Table 2:** Detailed information of hub areas in differentially covaried network of CHB.

	Involved pathway/function	Metabolites/Covariations	Change in CHB*	Significance**
Glycine metabolism	Glycine synthesis	Glycine(S)	0.68 (76.6%)	P = 0.00026
		Glycine(U)—Glycerate(S)	Decreased (0.55→-0.085)	Z = -3.33
	Phenolic compound metabolism	Glycine(U)—4-Hydroxyhippurate(U)	Increased (-0.11→0.49)	Z = 3.57
		Tyrosine(S)	1.43 (66.2%)	P = 0.01
	Bile acid synthesis	Glycine(S)—Cholesterol(S)	Increased (-0.27→0.34)	Z = 3.71
		Bile acid (Clinical indicator)	2.30 (54.6%)	P = 1.29E-05
	Entering into TCA cycle	Glycine(S)—Malic acid(S)	Increased (-0.58→0.19)	Z = -4.16
TCA cycle	Fatty acids~ TCA cycle	Succinate(S)—Oleic acid(S)	Decreased (0.70→0.007)	Z = -3.30
		Citrate(U)—Glycerol 1-octadecanoate(S)	Decreased (0.59→-0.026)	Z = -2.99
	Fatty acids	Hexadecanoic acid(S)	1.65 (75.9%)	P = 0.0019
		Oleic acid	1.61 (75.2%)	P = 0.0031
		Stearic acid(S)	1.43 (68.3%)	P = 0.0075

* For metabolites, the data is presented as the fold change (CHB/health) followed by proportion of CHB patients showing higher/lower level than the average value of healthy controls. For covariations, the data is presented as increased/decreased (*Cor*_*health*_ → *Cor*_*CHB*_).

** For metabolites, the significance was measured by P-value from two tailed student’s t test; and for covariations, Z-score was used instead. A significant difference between CHB group and healthy control exists if p-value < 0.05 or |Z| > 3.

#### Aberrant glycine metabolism in CHB

As Fig 2A indicated, glycine showed the highest degree of 23 in the differentially covaried network (S4 Table). By mapping to the known metabolic pathway, four bypasses related to glycine metabolism were suggested in Fig 2B: a) de novo synthesis of glycine, b) synthesis of conjugated bile acid, c) glycine transforming to hippurate and hydroxyhippurate and d) glycine entering TCA cycle.

1. Decreased glycine synthesis. In the pathway of glycine synthesis, covariation between glycerate and glycine was decreased from strong positive (0.55) in healthy group to extremely weak (-0.085) in CHB patients, suggesting a correlation loss between them. Comparing to healthy controls, decreased glycine in serum was detected in CHB samples (FC = 0.68, P = 0.00026), while the serumal glycerate remained unchanged in CHB group. To investigate whether this is caused by decreased synthesis, we further checked two available datasets of gene expression profile in CHB from GEO (S5 Table). One was from human PBMC (GSE58208) and the other was from liver tissues of woodchuck model (GSE36533 [14]). Among the 7 enzymes involved in transformation from glycerate to glycine, only one up-regulated gene was detected in PBMC of CHB patients. But in liver tissues of CHB woodchuck model, all the 7 genes were down regulated and 6 reached the significant level (P < 0.05, |logFC| > 0.5, S5 Table), suggesting decreased glycine synthesis in CHB liver.
2. Elevated bile acid synthesis. In bile acid synthesis, covariation between serumal glycine and serumal cholesterol was shifted from weak negative (-0.27) in healthy state to positive (0.34) in CHB patients (Z-score = 3.46). As we know, glycine is involved in the process of bile acid synthesis from cholesterol. Serological test showed significantly elevated bile acid in CHB group (FC = 2.30, P<<0.01), which was even higher in liver dysfunction subgroup than in patients with normal liver function (FC = 1.69, P = 0.037), as S6 Table indicated. Previous clinical observations showed increased conjugated bile acid as well in other HBV-induced liver diseases such as liver failure [16] and cirrhosis [17].
3. Increased hydroxyhippurate synthesis. Covariation between urinary 4-hydroxyhippurate and serumal glycine was shifted from weak negative (-0.11) in healthy individuals to positive (0.49) in CHB patients (|Z| = 3.57). Interestingly, when we divided CHB samples into subgroups of normal liver function and dysfunctional group, unchanged glycine was noticed in both subgroups and increased tendency of 4-hydroxyhippurate (FC = 1.07, P = 0.44) was observed with the increasing severity of liver dysfunction. Glycine is known to participate in the glycination of benzoic acid derivatives to form (hydroxy) hippurate, which helps to excrete phenolic compounds via urinary system. The positive covariation in CHB group and increased 4-hydroxyhippurate in liver dysfunction group suggested elevated excretion of phenolic compounds. This perfectly agreed with the widely accepted decreased ratio of branched chain amino acids to aromatic amino acids (BCAA/AAA) in previous reports about HBV related liver diseases [18]. In addition, tyrosine, a predominant AAA, was found to be increased in CHB patients’ serum (FC = 1.43, P = 0.01), which was consistent with increasing severity of liver dysfunction as well (S6 Table). The accumulation of aromatic metabolites might be a reason for the increased excretion of hydroxyhippurate in CHB patients.

So far, little evidence for abnormal glycine metabolism has been characterized for CHB, except one report of differentially changed glycine in HBV-induced liver failure [19]. Our results indicate significant changes in the biosynthesis and catabolism of glycine in CHB patients. Interestingly, protective effects of supplementing glycine have been verified in a rat model for alcohol-induced liver injury [20]. Moreover, treatment with glycine was shown to be beneficial to CHB patients [21].

#### Abnormal TCA cycle and fatty acid metabolism

Among the TOP 10 hub metabolites in S4 Table, two metabolites, malic acid and citrate, were involved in TCA cycle. In Fig 2C, covariations of three metabolic pairs were identified to be significantly differentially changed between CHB and healthy group. Among them, two are bridging TCA cycle with lipids (succinate ~ oleic acid and citrate ~ glycerol 1-octadecanoate). Moreover, both of the two covariations were disrupted from strong positive (0.70 and 0.59) in healthy volunteers to almost none (0.007 and -0.026) in CHB patients (|Z|> 3, Table 2), implying relation loss between TCA cycle and lipid metabolism. We examined the expression of enzyme-coding genes in metabolic network along the above two bridges, covering pathways of fatty acid (FA) biosynthesis/degradation and TCA cycle (S5 Table).

In FA synthesis, 2 and 6 differentially expressed genes were identified in PBMC and woodchuck model respectively (S5 Table). As a key lipogenic enzyme responsible for endogenous FA synthesis, fatty acid synthase (gene symbol: FASN) was found to be down regulated in liver tissue of CHB woodchuck model. However, Hu et al. reported consistent elevation of FASN in HBV-infected HepG2.2.15 cell, mice and HCC patients at protein level [22]. These conflicting observations may be related to the inherent gap between different molecular levels. Moreover, Lee et al. also observed elevation of downstream lipogenic genes (including FASN) in HBV-HCC patients [23]. They even verified that HBV could induce lipogenesis via activating LXRα in both cell culture and transgenic mouse model.

Among the 45 enzyme-coding genes in FA degradation, 2 up- and 11 down- regulated genes were found in PBMC of CHB patients. In the liver tissue of CHB woodchuck model, 22 were shown to be significantly changed and, more importantly, consistently down-regulated (S5 Table). It was hinted that the FA degradation might be depressed in CHB liver. Further, we compared the level of all serum FAs in different CHB stratifications. Hexadecanoic acid, oleic acid and stearic acid were found to be significantly increased in CHB patients (P < 0.05, Table 2). Moreover, their elevation was correlated with the increasing severity of liver dysfunction in CHB patients (P< 0.05, S6 Table).

In TCA cycle, 5 out of the 6 differentially expressed enzymes were found to be down-regulated in CHB woodchuck model. There are 3 rate-limiting enzymes in TCA cycle: citrate synthase (CS), α-ketoglutarate dehydrogenase (OGDH) and isocitrate dehydrogenase (IDH1 and IDH3A). In liver from CHB woodchuck model (see S5 Table), CS and IDH3A were up regulated whereas OGDH and IDH1 were down regulated (P < 0.05), although changes in OGDH and CS were mild (|logFC| < 0.5). As the overall metabolic flux may depend on rate-limiting enzymes with lowest activity or expression level, abnormal activity of TCA cycle was suggested in CHB. Similarly, abnormal enzyme expression in TCA cycle was observed in HBV-infected HepG2.2.15 cells [5]. Also, in HBV X protein (HBx) transfected hepatoma cells, it has been characterized that HBx could destroy the mitochondrial membrane potential [24] and cause mitochondrial dysregulation [25]. Then the relation between TCA metabolites and viral load was checked in CHB patients. A decrease of urinary citrate was detected in CHB subgroup with high viral copy (FC = 0.82, P = 0.034, S6 Table).

Although lipid metabolism disorder has been observed in HBV-infected cell [26] and animal model [27], our observations contributed new importance to the activity changes of abnormal TCA cycle and FA metabolism in CHB patients.

## Conclusions

It was recently highlighted that HBV infection can trigger metabolic reprogramming which is a hallmark of viral oncogenesis [28]. Here, to explore metabolic aberrations in CHB, we conducted a covariation analysis based on GC-MS metabolic profiles in serum and urine from CHB patients. Considering correlations are sensitive to sample size, two means were designed to reduce the bias: a) constructing randomizations by sampling from CHB patients and b) using relative ranking difference within respective groups. Functional indications in differentially co-varied metabolite pairs were interpreted by mapping to known pathways. Our results suggested reprogrammed pathways related to: a) glycine metabolism and b) bridge connecting fatty acids with TCA cycle in CHB patients. Whether these observations are specific to CHB patients or common in other HBV related liver diseases deserves further investigation.

Although the detected metabolites may be limited to the inherent ability of GC-MS, the present study could provide better insights into understanding the HBV-induced metabolic aberrations in CHB patients. With further improvement, the covariation analysis combined with network association study would pave an alternative but interesting way to interpret functional clues in clinical multi-omics data.

## Supporting Information

S1 Fig Intra- and inter- covariations in CHB population. (A) Intra- correlations between metabolites in serum; (B) Intra- correlations between metabolites in urine and (C) Inter- correlations across serumal and urinary metabolites.
(JPG)

S2 Fig Intra- and inter- covariations in healthy group. (A) Intra- correlations between metabolites in serum; (B) Intra- correlations between metabolites in urine; and (C) Inter- correlations across serumal and urinary metabolites.
(JPG)

S3 Fig Compositions of serum and urine metabolic profile. Categories were identified based on the chemical taxonomy in HMDB.
(JPG)

S1 Table Temperature program of column incubator in GC–MS. (DOCX)

S2 Table Identified metabolites in GC-MS profile of serum. (DOCX)

S3 Table Identified metabolites in GC-MS profile of urine. (DOCX)

S4 Table TOP 10 metabolites with high degree in differentially covaried metabolic network of CHB. (DOCX)

S5 Table Differentially expressed genes involved in TCA cycle, glycine biosynthesis and fatty acid metabolism. (DOCX)

S6 Table Changes of metabolites across CHB stratifications. (DOCX)
