# Supplementary figures and images for: Covariation Analysis of Serumal and Urinary Metabolites Suggests Aberrant Glycine and Fatty Acid Metabolism in Chronic Hepatitis B
Source: PLoS One. 2016 May 26;11(5):e0156166. doi: 10.1371/journal.pone.0156166 (PMC4881891; doi:10.1371/journal.pone.0156166)

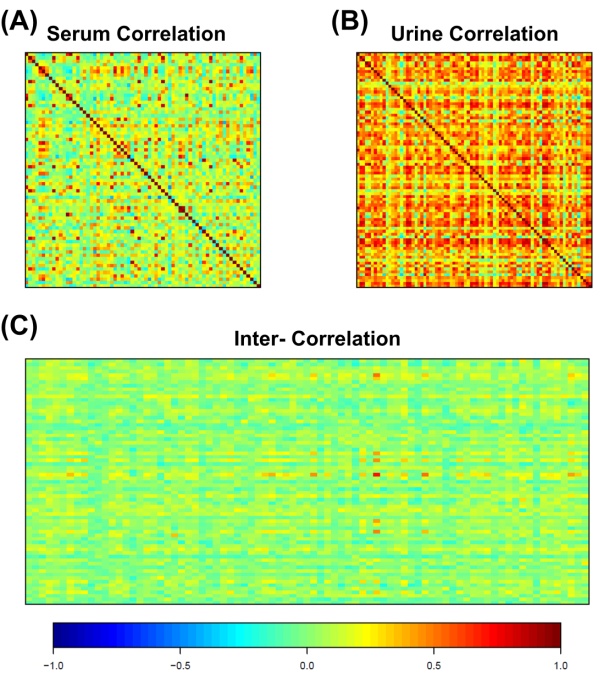

Supplement: S1 Fig — (A) Intra- correlations between metabolites in serum; (B) Intra- correlations between metabolites in urine and (C) Inter- correlations across serumal and urinary metabolites. (JPG) [file pone.0156166.s001.jpg]

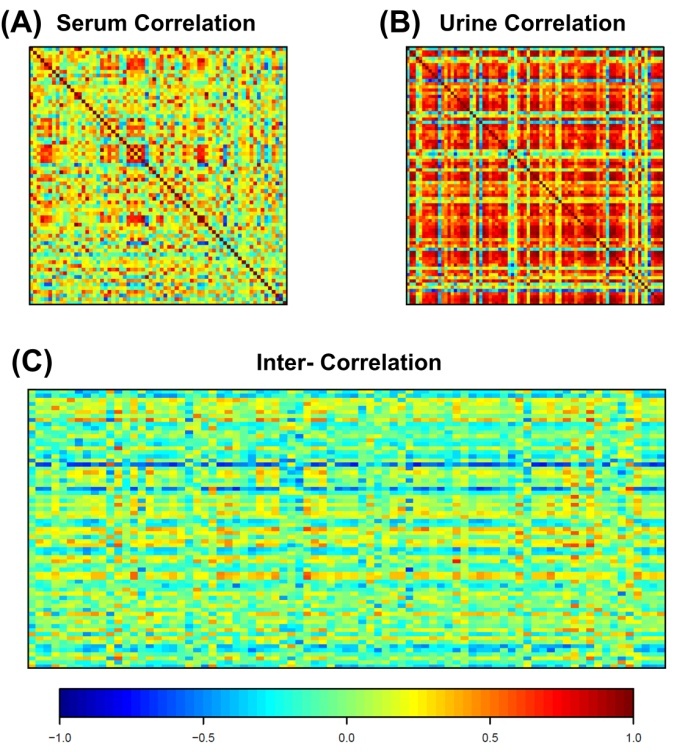

Supplement: S2 Fig — (A) Intra- correlations between metabolites in serum; (B) Intra- correlations between metabolites in urine; and (C) Inter- correlations across serumal and urinary metabolites. (JPG) [file pone.0156166.s002.jpg]

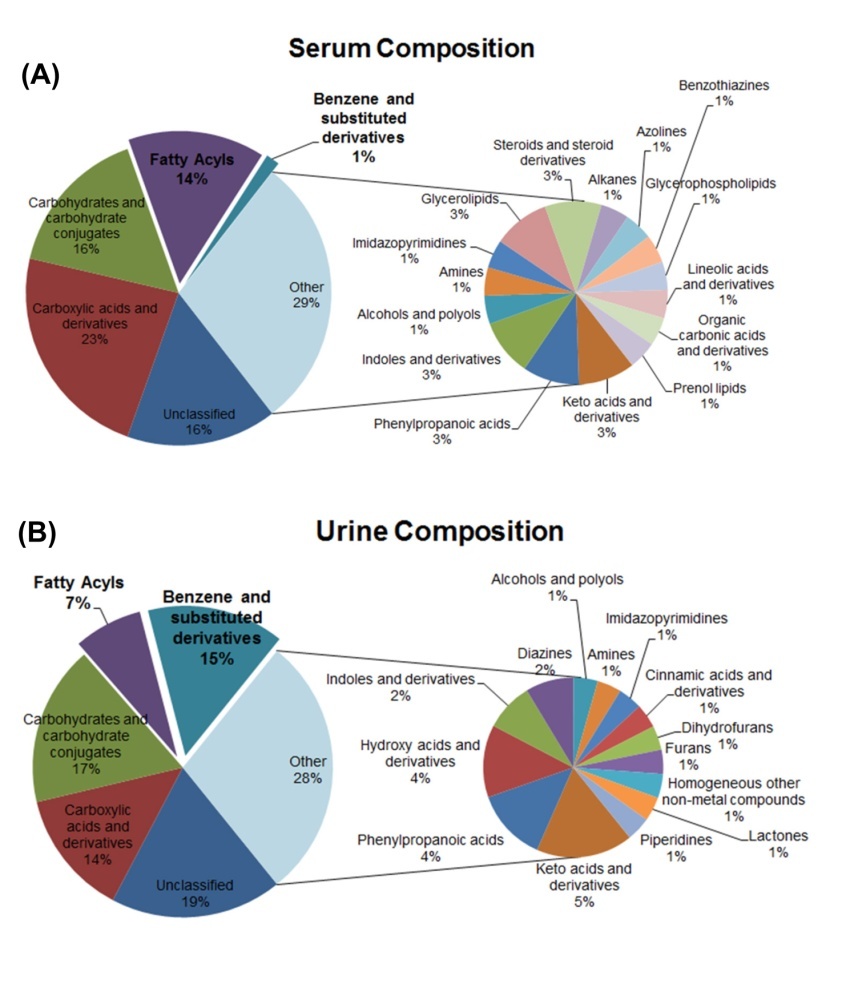

Supplement: S3 Fig — Categories were identified based on the chemical taxonomy in HMDB. (JPG) [file pone.0156166.s003.jpg]
