# Supplementary material for: Covariation Analysis of Serumal and Urinary Metabolites Suggests Aberrant Glycine and Fatty Acid Metabolism in Chronic Hepatitis B
Source: PLoS One. 2016 May 26;11(5):e0156166. doi: 10.1371/journal.pone.0156166 (PMC4881891; doi:10.1371/journal.pone.0156166)
Supplement: S1 Table — (DOCX) [file pone.0156166.s004.docx]

**S1 Table. Temperature program of column incubator in GC - MS**

| **Project** | **Rate(℃/min)** | **Temperature(℃)** | **Hold time(min)** |
| --- | --- | --- | --- |
| Urine |  | 70 | 2 |
|  | 2.5 | 160 | 0 |
|  | 5 | 240 | 16 |
| Serum |  | 80 | 2 |
|  | 5 | 240 | 0 |
|  | 25 | 290 | 10 |
