# Supplementary material for: Covariation Analysis of Serumal and Urinary Metabolites Suggests Aberrant Glycine and Fatty Acid Metabolism in Chronic Hepatitis B
Source: PLoS One. 2016 May 26;11(5):e0156166. doi: 10.1371/journal.pone.0156166 (PMC4881891; doi:10.1371/journal.pone.0156166)
Supplement: S2 Table — (DOCX) [file pone.0156166.s005.docx]

**S2 Table. Identified metabolites in GC-MS profile of serum**

| Retention time (min) | Qual(%) | Postulated chemicals | HMDB ID | KEGG ID |
| --- | --- | --- | --- | --- |
| 22.02 | 99 | 9,12-Octadecadienoic acid | HMDB00673 | C01595 |
| 6.42 | 91 | 2-Hydroxybutyric acid | HMDB00008 | C05984 |
| 31.56 | 99 | Cholesterol | HMDB00067 | C00187 |
| 8.93 | 96 | Glycine | HMDB00123 | C00037 |
| 14.85 | 94 | 3-Glycerophosphate | HMDB00126 | C00093 |
| 9.21 | 96 | Glycerate | HMDB00139 | C00258 |
| 18.18 | 94 | alpha.-D-Galactopyranose | HMDB00143 | C00984 |
| 11.28 | 91 | Malic acid | HMDB00156 | C00149 |
| 15.57 | 99 | Purine-6-ol | HMDB00157 | C00262 |
| 17.58 | 91 | tyrosine | HMDB00158 | C00082 |
| 13.05 | 91 | L-phenylalanine | HMDB00159 | C00079 |
| 6.12 | 97 | l-Alanine | HMDB00161 | C00041 |
| 8.83 | 94 | L-Proline | HMDB00162 | C00148 |
| 9.96 | 91 | L-threonine | HMDB00167 | C00188 |
| 13.59 | 99 | L-Asparagine | HMDB00168 | C00152 |
| 18.19 | 93 | D-Mannose | HMDB00169 | C00159 |
| 8.74 | 90 | L-Isoleucine | HMDB00172 | C00407 |
| 17.34 | 96 | L-Lysine | HMDB00182 | C00047 |
| 11.70 | 95 | L-Aspartic acid | HMDB00191 | C00049 |
| 22.14 | 99 | Oleic acid | HMDB00207 | C00712 |
| 12.38 | 93 | 2-oxo-Pentanedioic acid(Oxoglutaric acid) | HMDB00208 | C00026 |
| 19.95 | 91 | Myo-Inositol | HMDB00211 | C00137 |
| 15.69 | 99 | L-Ornithine | HMDB00214 | C00077 |
| 19.35 | 98 | Hexadecanoic acid | HMDB00220 | C00249 |
| 5.55 | 91 | 2-Oxopropanoate | HMDB00243 | C00022 |
| 9.03 | 98 | Butanedioic acid | HMDB00254 | C00042 |
| 11.74 | 90 | 5-hydroxo-L-Proline | HMDB00267 | C01879 |
| 8.21 | 95 | Urea | HMDB00294 | C00086 |
| 9.69 | 80 | 1H-Indole-3-ethanamine | HMDB00303 | C00398 |
| 15.39 | 93 | Ribitol | HMDB00508 | C00474 |
| 17.84 | 90 | D-Glucopyranose | HMDB00516 | C00221 |
| 12.13 | 95 | Creatinine | HMDB00562 | C00791 |
| 14.23 | 93 | Arabinitol | HMDB00568 | C01904 |
| 12.95 | 95 | Glutamine | HMDB00641 | C00064 |
| 8.14 | 90 | Propanedioic acid | HMDB00691 | C00383 |
| 11.73 | 97 | 2-pyrrolidone carboxylic acid | HMDB00805 | C02237 |
| 22.55 | 99 | Octadecanoic acid | HMDB00827 | C01530 |
| 7.67 | 97 | L-Valine | HMDB00883 | C00183 |
| 21.80 | 96 | Tryptophan | HMDB00929 | C00078 |
| 24.42 | 95 | Arachidonic acid | HMDB01043 | C00219 |
| 11.07 | 91 | Aminomalonic acid | HMDB01147 | C00872 |
| 31.32 | 99 | alpha-Tocopherol (vitamin E) | HMDB01893 | C02477 |
| 25.24 | 99 | Eicosanoic acid | HMDB02212 | C06425 |
| 11.36 | 80 | Cadaverine | HMDB02322 | C01672 |
| 18.20 | 91 | beta.-d-Galactopyranose | HMDB03449 | C00124 |
| 11.77 | 91 | 4-hydroxy-L-Proline | HMDB06055 | C01157 |
| 17.08 | 83 | 2-Deoxy-galactopyranose | HMDB12327 | C02781 |
| 18.95 | 95 | Palmitelaidic acid | HMDB12328 |  |
| 6.90 | 95 | 4-hydroxy-Butanoic acid | HMDB15507 | C00989 |
| 26.29 | 90 | 2,3-Dihydroxypropyl hexadecanoate | HMDB31074 | |
| 27.64 | 83 | monostearin | HMDB31075 | |
| 5.24 | 80 | 1,3,5-trimethyl-Benzene | HMDB41924 | C14508 |
| 26.41 | 98 | Eicosane | HMDB59909 |  |
| 12.85 | 90 | alpha.-D-Arabinopyranose | | C00216 |
| 12.04 | 90 | (5.alpha.)-Androstane | | C01554 |
| 23.94 | 87 | Pseudouridine | | C02067 |
| 15.12 | 83 | alpha.-D-Fructofuranoside | | C03437 |
| 22.12 | 92 | 4-nitro-2-diphenylphosphino-Phenol | | |
| 12.95 | 93 | 6-hydroxy-2-aminohexanoic acid | | |
| 16.07 | 86 | 1,2-O-Isopropylidene-D-glucofuranose | | |
| 13.66 | 91 | Fructose oxime | |  |
| 17.22 | 93 | Galactose oxime | |  |
| 13.94 | 90 | 5-Phenyl-2-phenylthio-cyclohexanone | | |
| 17.22 | 91 | o-methyloxyme, (1Z)-d-Glucose | | |
| 7.40 | 83 | 2-hydroxy-2-Butenoic acid | | |
| 16.91 | 91 | o-methyloxyme, (1E)-d-Glucose | | |
| 11.95 | 93 | 2,3,4-Trihydroxybutyric acid | | |
| 8.68 | 87 | Butane |  |  |
| 22.13 | 92 | 10H-Phenothiazine,  10-[3-(4-methyl-1-piperazinyl)propyl]- | | |
