# Supplementary material for: Covariation Analysis of Serumal and Urinary Metabolites Suggests Aberrant Glycine and Fatty Acid Metabolism in Chronic Hepatitis B
Source: PLoS One. 2016 May 26;11(5):e0156166. doi: 10.1371/journal.pone.0156166 (PMC4881891; doi:10.1371/journal.pone.0156166)
Supplement: S3 Table — (DOCX) [file pone.0156166.s006.docx]

**S3 Table. Identified metabolites in GC-MS profile of urine**

| Retention time (min) | Qual(%) | Postulated chemicals | HMDB ID | KEGG ID |
| --- | --- | --- | --- | --- |
| 44.41 | 90 | (3-Hydroxy-4-methoxyphenyl)ethylene glycol | HMDB00559 | |
| 40.48 | 99 | 2,6-Dihydroxy-4-pyrimidinecarboxylic acid | HMDB00226 | C00295 |
| 12.11 | 90 | 3-methyl-2-oxo-Butanoic acid | HMDB00019 | C00141 |
| 46.22 | 91 | L-Ascorbic acid | HMDB00044 | C00072 |
| 24.48 | 86 | Acetoacetic acid | HMDB00060 | C00164 |
| 43.14 | 99 | 2-Hydroxy-1,2,3-propanetricarboxylic acid | HMDB00094 | C00158 |
| 45.90 | 93 | Galactitol | HMDB00107 | C01697 |
| 10.09 | 91 | hydroxy-Acetic acid | HMDB00115 | C00160 |
| 20.40 | 95 | Glycine | HMDB00123 | C00037 |
| 18.87 | 93 | Glycerol | HMDB00131 | C00116 |
| 47.87 | 98 | Purine-6-ol | HMDB00157 | C00262 |
| 44.67 | 95 | Tyrosine | HMDB00158 | C00082 |
| 35.55 | 95 | phenylalanine | HMDB00159 | C00079 |
| 19.94 | 95 | l-Threonine | HMDB00167 | C00188 |
| 45.62 | 98 | L-Lysine | HMDB00182 | C00047 |
| 9.43 | 91 | 2-hydroxy-Propanoic acid | HMDB00190 | C00186 |
| 51.63 | 95 | Oleic acid | HMDB00207 | C00712 |
| 46.48 | 92 | myo-Inositol | HMDB00211 | C00137 |
| 45.83 | 91 | glucitol | HMDB00247 | C00794 |
| 20.73 | 98 | Butanedioic acid | HMDB00254 | C00042 |
| 46.05 | 99 | 3,4-Dihydroxy hydrocinnamic acid | HMDB00423 | C10447 |
| 41.23 | 99 | Vanillic acid | HMDB00484 | C06672 |
| 23.30 | 95 | N-acetyl-Glycine | HMDB00532 |  |
| 39.99 | 91 | Arabitol | HMDB00568 | C01904 |
| 32.25 | 93 | 2-amino-3-mercapto-propionic acid | HMDB00574 | C00097 |
| 39.07 | 87 | alpha.-D-Arabinopyranose | HMDB00646 | C00259 |
| 47.53 | 91 | Glucaric acid | HMDB00663 | C00818 |
| 32.49 | 94 | o-Hydroxyphenylacetic acid | HMDB00669 | C05852 |
| 12.76 | 94 | 3-hydroxy-Propanoic acid | HMDB00700 | C01013 |
| 43.44 | 90 | N-benzoyl-Glycine | HMDB00714 | C01586 |
| 51.44 | 91 | 5-hydroxy indoleacetic acid | HMDB00763 | C05635 |
| 45.92 | 94 | D-Mannitol | HMDB00765 | C00392 |
| 30.45 | 94 | 2-pyrrolidone carboxylic acid | HMDB00805 | C02237 |
| 41.96 | 93 | Ribonic acid | HMDB00867 | C01685 |
| 51.43 | 98 | L-Tryptophan | HMDB00929 | C00078 |
| 49.17 | 99 | Ferulic acid | HMDB00954 | C01494 |
| 36.21 | 91 | 2,3-hydroxy-Succinic acid | HMDB00956 | C00898 |
| 41.07 | 91 | 1-Propene-1,2,3-tricarboxylic acid | HMDB00958 | C02341 |
| 38.37 | 91 | Xylulose | HMDB01644 | C00310 |
| 43.02 | 97 | 3,4-hydroxy-Benzoic acid | HMDB01856 | C00230 |
| 45.03 | 80 | 6-amino-Hexanoic acid | HMDB01901 | C02378 |
| 21.06 | 87 | 2-methyl-Alanine | HMDB01906 | C03665 |
| 19.03 | 86 | dimethyl-Propanedioic acid | HMDB02001 | |
| 22.37 | 86 | methylene-Butanedioic acid | HMDB02092 | C00490 |
| 36.24 | 89 | 1,2-Benzenedicarboxylic acid, ethyl ester | HMDB02120 | C01606 |
| 52.37 | 97 | Eicosanoic acid | HMDB02212 | C06425 |
| 27.63 | 90 | Cadaverine | HMDB02322 | C01672 |
| 11.21 | 99 | hydroxylamine | HMDB03338 | C00192 |
| 44.53 | 90 | L-Gluconic acid | HMDB03466 | C01040 |
| 15.84 | 97 | beta.-Amino isobutyric acid | HMDB03911 | C05145 |
| 36.85 | 95 | 2,5-Furandicarboxylic acid | HMDB04812 | C20450 |
| 50.48 | 97 | 3-Hydroxyhippuric acid | HMDB06116 | |
| 51.73 | 98 | N-(4-hydroxybenzoyl)-Glycine | HMDB13678 |  |
| 6.62 | 95 | Benzene, 1,2,4-trimethyl- | HMDB13733 | C14533 |
| 6.46 | 87 | Benzene, (1-methylethyl)- | HMDB34029 | C14396 |
| 6.13 | 90 | Piperidine | HMDB34301 | C01746 |
| 54.67 | 90 | Octadecanoic acid, butyl ester | HMDB40290 |  |
| 6.50 | 92 | 1-ethyl-4-methyl-Benzene | HMDB59832 | |
| 6.51 | 97 | 1-ethyl-3-methyl-Benzene | HMDB59848 | C14522 |
| 61.20 | 91 | alpha.-D-Glucopyranoside | HMDB61922 |  |
| 43.28 | 98 | 3,4-Dihydroxymandelate | | C05580 |
| 44.95 | 93 | Vanillylpropionic acid |  |  |
| 45.70 | 91 | Sedoheptulose, o-methyloxime |  |  |
| 13.58 | 91 | Propanoic acid, 2-methyl-3-hydroxy- |  |  |
| 43.85 | 99 | O-methyloxime-D-Fructose |  |  |
| 45.07 | 86 | N,N-bis(2-ethyl)ethanamine |  |  |
| 37.46 | 91 | Fructose oxime |  |  |
| 41.38 | 92 | 6-deoxy-D-Glucitol |  |  |
| 11.74 | 86 | 4-hydroxy-Pentenoic acid |  |  |
| 35.70 | 81 | 3-deoxy-D-Ribo-Hexitol |  |  |
| 53.61 | 94 | 2-O-Glycerol-.alpha.-d-galactopyranoside |  |  |
| 8.54 | 91 | 2-Methyl-1,3-bis(hydroxy)butane |  |  |
| 37.14 | 90 | 2-hydroxy-2-Pentenoic acid |  |  |
| 41.85 | 90 | 2-hydroxy-2-Butenoic acid |  |  |
| 44.04 | 91 | 2,4-hydroxy-Pentanedioic aci |  |  |
| 55.58 | 95 | 2,4,6(1H,3H,5H)-Pyrimidinetrione, 5-[2,3-dihydroxy-2-propenyl]-1,3-dimethyl-5-(1-methylbutyl)- |  | |
| 27.13 | 90 | O,O,O-Triethyl thiophosphate | | |
| 35.79 | 90 | 2,5-Dimethyldiphenylsulfone | | |
| 13.06 | 99 | Dihydroxy-ammonia | | |
| 8.96 | 94 | 2-(methoxyimino)-Propanoic acid | | |
| 33.81 | 94 | 2-(methoxyimino)-Pentanedioic acid | | |
