# Supplementary material for: Covariation Analysis of Serumal and Urinary Metabolites Suggests Aberrant Glycine and Fatty Acid Metabolism in Chronic Hepatitis B
Source: PLoS One. 2016 May 26;11(5):e0156166. doi: 10.1371/journal.pone.0156166 (PMC4881891; doi:10.1371/journal.pone.0156166)
Supplement: S4 Table — (DOCX) [file pone.0156166.s007.docx]

**S4 Table. TOP 10 metabolites with high degree in differentially covaried metabolic network of CHB**

| **Rank*** | **Degree** | **Source** | **Name** | **HMDB ID** | **KEGG ID** |
| --- | --- | --- | --- | --- | --- |
| 1 | 23 | urine | Glycine | HMDB00123 | C00037 |
| 2 | 19 | urine | 2-(methoxyimino)-Pentanedioic acid | HMDB00208 | C00026 |
| 3 | 18 | urine | Oleic acid | HMDB00207 | C00712 |
| 4 | 16 | urine | Sedoheptulose | HMDB03219 | C02076 |
| 5 | 13 | urine | 4-hydroxy-Pentenoic acid | - | - |
| 6 | 9 | urine | Glycerol | HMDB00131 | C00116 |
| 7 | 6 | urine | Malic acid | HMDB00156 | C00149 |
| 8 | 5 | serum | Piperidine | HMDB34301 | C01746 |
| 9 | 4 | urine | Citrate | HMDB00094 | C00158 |
| 9 | 4 | urine | methylene-Butanedioic acid | HMDB02092 | C00490 |
| 9 | 4 | urine | 3,4-hydroxy-Benzoic acid | HMDB01856 | C00230 |
| 9 | 4 | serum | Glycine | HMDB00123 | C00037 |
| 9 | 4 | urine | Ribonic acid | HMDB00867 | C01685 |
| 9 | 4 | urine | Ferulic acid | HMDB00954 | C01494 |
| 9 | 4 | urine | Vanillic acid | HMDB00484 | C06672 |
| 9 | 4 | urine | Fructose | HMDB00660 | C02336 |
| 9 | 4 | urine | L-Gluconic acid | HMDB03466 | C01040 |
| 9 | 4 | urine | 3,4-Dihydroxymandelate | | C05580 |
| 9 | 4 | urine | 1-Propene-1,2,3-tricarboxylic acid | HMDB00958 | C02341 |

*Because there were 11 compounds tied for the 9^th^ place, there were 19 metabolites in TOP 10 list.
