# Supplementary material for: Covariation Analysis of Serumal and Urinary Metabolites Suggests Aberrant Glycine and Fatty Acid Metabolism in Chronic Hepatitis B
Source: PLoS One. 2016 May 26;11(5):e0156166. doi: 10.1371/journal.pone.0156166 (PMC4881891; doi:10.1371/journal.pone.0156166)
Supplement: S5 Table — (DOCX) [file pone.0156166.s008.docx]

**S5 Table. Differentially expressed genes involved in TCA cycle, glycine biosynthesis and fatty acid metabolism**

| **Gene symbol** | **Gene ID** | **CHB woodchuck** | | | **CHB PBMC** | | |
| --- | --- | --- | --- | --- | --- | --- | --- |
|  |  | **Up/Down** | **logFC** | **P-value** | **Up/Down** | **logFC** | **P-value** |
| **Enzymes related to TCA cycle** | | | | | | | |
| SUCLG2 | 8801 | down | -0.55 | 0.00 | down | -0.72 | 0.00 |
| DLAT | 1737 |  |  |  | down | -0.70 | 0.00 |
| DLD | 1738 |  |  |  | down | -0.91 | 0.00 |
| FH | 2271 |  |  |  | down | -1.18 | 0.01 |
| PDHB | 5162 |  |  |  | down | -0.89 | 0.00 |
| SDHB | 6390 |  |  |  | down | -0.70 | 0.00 |
| SDHC | 6391 |  |  |  | down | -0.89 | 0.02 |
| SDHD | 6392 |  |  |  | down | -0.75 | 0.00 |
| SUCLA2 | 8803 |  |  |  | down | -0.82 | 0.00 |
| ACLY | 47 |  |  |  | up | 0.66 | 0.01 |
| ACO2 | 50 |  |  |  | up | 0.86 | 0.00 |
| IDH2 | 3418 |  |  |  | up | 0.89 | 0.00 |
| IDH3G | 3421 |  |  |  | up | 0.72 | 0.01 |
| *OGDH | 4967 |  | -0.15 | 0.01 | up | 1.79 | 0.00 |
| *CS | 1431 |  | 0.16 | 0.01 |  | -0.03 | 0.67 |
| ACO1 | 48 | down | -1.12 | 0.00 |  |  |  |
| *IDH1 | 3417 | down | -0.50 | 0.00 |  |  |  |
| PCK1 | 5105 | down | -1.52 | 0.00 |  |  |  |
| SDHA | 6389 | down | -0.53 | 0.04 |  |  |  |
| *IDH3A | 3419 | up | 0.94 | 0.00 |  |  |  |
| **Enzymes in glycine biosynthesis** | | | | | | | |
| SHMT2 | 6472 | down | -0.66 | 0.02 | up | 1.33 | 0.00 |
| AGXT | 189 | down | -1.91 | 0.00 |  |  |  |
| GLYCTK | 132158 | down | -0.77 | 0.00 |  |  |  |
| PHGDH | 26227 | down | -0.82 | 0.00 |  |  |  |
| PSAT1 | 29968 | down | -0.69 | 0.00 |  |  |  |
| SHMT1 | 6470 | down | -0.54 | 0.00 |  |  |  |
| **Enzymes in fatty acid biosynthesis** | | | | | | | |
| ACACA | 31 | down | -1.53 |  |  |  |  |
| ACACB | 32 | down | -1.86 |  |  |  |  |
| FASN | 2194 | down | -2.57 |  |  |  |  |
| ^§^ACSL6 | 23305 |  |  |  | down | -0.93 | 0.02 |
| ^§^ACSL4 | 2182 | down | -0.95 | 0.00 | up | 0.67 | 0.03 |
| ^§^ACSL1 | 2180 | down | -0.67 | 0.00 |  |  |  |
| ^§^ACSL3 | 2181 | down | -0.73 | 0.00 |  |  |  |
| **Enzymes in fatty acid degradation** | | | | | | | |
| ACAA2 | 10449 | down | -0.63 | 0.00 | down | -0.55 | 0.02 |
| ACADM | 34 | down | -0.75 | 0.00 | down | -0.60 | 0.00 |
| ACADSB | 36 | down | -1.31 | 0.00 | down | -0.98 | 0.00 |
| ADH5 | 128 | down | -0.51 | 0.00 | down | -0.95 | 0.00 |
| ALDH2 | 217 | down | -1.11 | 0.00 | down | -0.74 | 0.01 |
| ALDH3A2 | 224 | down | -0.56 | 0.00 | down | -0.84 | 0.00 |
| ACAT2 | 39 |  |  |  | down | -0.67 | 0.00 |
| ECI2 | 10455 |  |  |  | down | -0.65 | 0.00 |
| HADH | 3033 |  |  |  | down | -0.51 | 0.03 |
| HADHA | 3030 |  |  |  | down | -0.75 | 0.00 |
| ACADVL | 37 |  |  |  | up | 1.57 | 0.00 |
| ACAA1 | 30 | down | -0.88 | 0.00 |  |  |  |
| ACADS | 35 | down | -0.90 | 0.00 |  |  |  |
| ACOX1 | 51 | down | -0.54 | 0.00 |  |  |  |
| ADH1C | 126 | down | -1.97 | 0.00 |  |  |  |
| ADH4 | 127 | down | -1.55 | 0.00 |  |  |  |
| ADH6 | 130 | down | -0.51 | 0.02 |  |  |  |
| ALDH1B1 | 219 | down | -1.07 | 0.00 |  |  |  |
| ALDH7A1 | 501 | down | -0.68 | 0.00 |  |  |  |
| CPT1A | 1374 | down | -0.52 | 0.01 |  |  |  |
| CYP4A11 | 1579 | down | -0.61 | 0.00 |  |  |  |
| CYP4A22 | 284541 | down | -0.97 | 0.00 |  |  |  |
| EHHADH | 1962 | down | -0.96 | 0.00 |  |  |  |
| GCDH | 2639 | down | -0.82 | 0.00 |  |  |  |

^§^These genes are shared by pathways of fatty acid biosynthesis and degradation.

*These genes are rate-limiting enzymes in TCA cycle.
