# Supplementary material for: Covariation Analysis of Serumal and Urinary Metabolites Suggests Aberrant Glycine and Fatty Acid Metabolism in Chronic Hepatitis B
Source: PLoS One. 2016 May 26;11(5):e0156166. doi: 10.1371/journal.pone.0156166 (PMC4881891; doi:10.1371/journal.pone.0156166)
Supplement: S6 Table — (DOCX) [file pone.0156166.s009.docx]

**S6 Table. Changes of metabolites across CHB stratifications**

| **Name** | **Source** | **Annotation** | **CHB v.s. Health*** | **Changes between CHB subgroups** | |
| --- | --- | --- | --- | --- | --- |
|  |  |  |  | **Liver dysfunction vs. Normal function*** | **High viral load v.s. low viral load*** |
| Glycine | Serum | other | 0.68 (0.000) | - | 1.39 (0.006) |
| Glycine | Urine | other | - | - | - |
| 4-hydroxy-hypurate | Urine | other | - | 1.07 (0.44) | - |
| Cholesterol | Serum | other | - | - | - |
| Glycerate | Serum | other | - | - | - |
| Tyrosine | Serum | Aromatic amino acid | 1.43 (0.011) | - | 0.79 (0.031) |
| Stearic acid | Serum | Fatty acid | 1.43 (0.008) | 1.27 (0.025) | 0.81 (0.031) |
| Hexadecanoic acid | Serum | Fatty acid | 1.65 (0.002) | 1.35 (0.010) | - |
| Oleic acid | Serum | Fatty acid | 1.61 (0.003) | 1.31 (0.031) | - |
| Malic acid | Serum | TCA | ↑(0.061) | - | ↓(0.009) |
| Citrate | Urine | TCA | - | - | 0.82 (0.034) |
| Succinate | Serum | TCA | - | - | - |
| Succinate | Urine | TCA | - | - | - |
| Total bile acid | Serological test | Clinical Idicator | 2.30 (0.000) | 1.69 (0.037) | - |
| Glycine | Serum | other | 0.68 (0.000) | - | 1.39 (0.006) |

*Data was represented as fold change followed by p-values between groups (two tailed student’s t test).
